# IL‐7 suppresses macrophage autophagy and promotes liver pathology in *Schistosoma japonicum*‐infected mice

**DOI:** 10.1111/jcmm.13610

**Published:** 2018-03-22

**Authors:** Jifeng Zhu, Weiwei Zhang, Lina Zhang, Lei Xu, Xiaojun Chen, Sha Zhou, Zhipeng Xu, Ming Xiao, Hui Bai, Feng Liu, Chuan Su

**Affiliations:** ^1^ Department of Pathogen Biology and Immunology Jiangsu Key Laboratory of Pathogen Biology Nanjing Medical University Jiangning District, Nanjing Jiangsu China; ^2^ Department of Pathogen Biology Nanjing University of Chinese Medicine Nanjing Jiangsu China; ^3^ Department of Anatomy Nanjing Medical University Jiangning District, Nanjing Jiangsu China; ^4^ Department of Pathology and Physiology Atherosclerosis Research Center Key Laboratory of Cardiovascular Disease and Molecular Intervention Nanjing Medical University Jiangning District, Nanjing Jiangsu China

**Keywords:** IL‐7, liver immunopathology, macrophage autophagy, Schistosomiasis

## Abstract

In schistosomiasis japonica and mansoni, parasite eggs trapped in host liver elicit severe liver granulomatous inflammation that subsequently leads to periportal fibrosis, portal hypertension, haemorrhage or even death. Macrophages are critical for granuloma formation and the development of liver fibrosis during schistosomiasis. However, whether the aberrant regulation of macrophage autophagy has an effect on the development of liver immunopathology in schistosomiasis remains to be elucidated. In this study, we showed that *Schistosoma japonicum* (*S. japonicum*) egg antigen (SEA)‐triggered macrophage autophagy limited the development of pathology in host liver. However, engagement of IL‐7 receptor (IL‐7R/CD127) on macrophages by *S. japonicum* infection‐induced IL‐7 significantly suppressed SEA‐triggered macrophage autophagy, which led to an enhanced liver pathology. In addition, anti‐IL‐7 neutralizing antibody or anti‐CD127 blocking antibody treatment increased macrophage autophagy and suppressed liver pathology. Finally, we demonstrated that IL‐7 protects macrophage against SEA‐induced autophagy through activation of AMP‐activated protein kinase (AMPK). Our study reveals a novel role for IL‐7 in macrophage autophagy and identifies AMPK as a novel downstream mediator of IL‐7‐IL‐7R signalling and suggests that manipulation of macrophage autophagy by targeting IL‐7‐IL‐7R signalling may have the potential to lead to improved treatment options for liver pathogenesis in schistosomiasis.

## 1 INTRODUCTION

Schistosomiasis is a parasitic disease affecting more than 210 million people worldwide.^1^ Infection of *Schistosoma japonicum* (*S. japonicum*) or *Schistosoma mansoni* (*S. mansoni*) leads to severe liver granulomatous immunopathology induced by eggs trapped in the liver.^2^, ^3^ The granulomatous response is characterized by large influxes of inflammatory cells, particularly macrophages, which compose up to 30% of granuloma cells.^4^, ^5^ In addition, as one of the major types of antigen‐presenting cells (APC), macrophages also play a critical role in the initiation and regulation of granulomatous inflammation by presenting schistosome egg antigens to CD4^+^ T cells to secrete a variety of cytokines and chemokines.^2^, ^5^, ^6^ These factors recruit and activate more macrophages and other inflammatory cells including eosinophils, neutrophils and mononuclear cells to the liver to form granulomas by enveloping the eggs.^5^ Moreover, granuloma macrophages mediate collagen synthesis through a variety of mechanisms and finally result in periportal fibrosis, portal hypertension, haemorrhage or even death.^2^, ^7^, ^8^

Autophagy of the macrophages has a critical regulatory function in down‐regulation of liver immunopathology in various diseases. For example, macrophage autophagy functions to limit liver inflammation in D‐galactosamine/lipopolysaccharide (GalN/LPS)‐treated mice by inhibiting the generation of inflammasome‐dependent IL‐1β.^9^ In high‐fat diet‐fed, LPS‐treated mice, the loss of macrophage autophagy promoted liver inflammation.^10^ Macrophage autophagy deficiency not only enhances infiltration of the liver by inflammatory cells, but exacerbates liver fibrosis by enhancing the fibrogenic properties of hepatic myofibroblasts via an IL‐1‐dependent pathway in mice exposed to CCl4.^11^ However, given that macrophage plays critical role in schistosome egg‐triggered immunopathology in host liver, little is known about whether and how aberrant regulation of macrophage autophagy can impact the liver immunopathology after schistosome infection.

Pro‐inflammatory cytokine IL‐7 is a “stromal cytokine” produced in the liver, bone marrow, lymph node, skin and gut.^12^, ^13^, ^14^ IL‐7 exerts a variety of effects by binding to the IL‐7 receptor (IL‐7R), consisting of IL‐7Rα (CD127) and γc (CD132).^14^ Although the underline mechanisms are still unknown, the role of IL‐7 in modulating immunopathology during schistosome infection has been reported more than a dozen years ago. IL‐7 deficiency markedly reduced level of collagen in the livers of *S. mansoni*‐infected mice.^15^ Reversely, *S. mansoni*‐infected IL‐7 transgenic mice showed aggravated collagen deposition.^16^

Recently, study suggests that IL‐7 may be involved in the autophagic process. In a T‐cell line, D1, deprivation of IL‐7 caused an increased number of autophagosomes, suggesting a possible anti‐autophagic role of IL‐7 in T cells.^17^ However, whether IL‐7 can also regulate macrophage autophagy and subsequently impact on the tissue immunopathology has never been reported.

In this study, we demonstrated that IL‐7 suppressed macrophage autophagy and enhanced liver immunopathology in *S. japonicum*‐infected mice. The effect of IL‐7 on liver immunopathology was mediated by the inhibition of AMP‐activated protein kinase (AMPK)‐dependent macrophage autophagy. Our results suggest that induction of macrophage autophagy through IL‐7 blockade may be a potential therapeutic strategy against tissue immunopathology in diseases including schistosomiasis.

## 2 MATERIALS AND METHODS

### 2.1 Ethics statement

Animal experiments were performed in strict accordance with the Regulations for the Administration of Affairs Concerning Experimental Animals (1988.11.1), and all efforts were made to minimize suffering. All animal procedures were approved by the Institutional Animal Care and Use Committee (IACUC) of Nanjing Medical University for the use of laboratory animals (Permit Number: NJMU 20121110).

### 2.2 Mice infection and antigen preparation

*Oncomelania hupensis* harbouring *S. japonicum* cercariae (Chinese mainland strain) were purchased from the Parasitic Disease Prevention and Research Institute of Jiangsu Province. Eight‐week‐old female C57BL/6 mice (SLAC Laboratory, Shanghai, China) were infected with 12 cercariae of *S. japonicum* through the abdominal skin.

Soluble egg antigen (SEA) was prepared from purified and homogenized *S. japonicum* eggs. The protein concentration of SEA was determined by the bicinchoninic acid (BCA) Protein Assay kit (Bio‐Rad, Hercules, CA).

### 2.3 Cytokine and antibody treatment of infected mice

Three independent experiments were carried out. In each experiment, 36 *S. japonicum*‐infected mice were randomly assigned in six groups (six mice per group) to receive injections of either cytokine, antibody or their control solutions 3 weeks after *S. japonicum* infection. Mice were killed for further experiment 6 weeks after infection.

For cytokine treatment, 2.5 μg recombinant murine IL‐7 (Peprotech, Rocky Hill, NJ) in 200 μL PBS or 200 μL PBS alone per mouse was intraperitoneally (i.p.) injected. Injections were repeated every other day until 2 days before the mice were killed.

For antibody treatment, 100 μg goat anti‐mouse IL‐7 neutralizing antibody (R&D Systems, Minneapolis, MN) or 100 μg goat IgG isotype control antibody (R&D Systems) or 200 μg rat anti‐mouse CD127 (IL‐7 receptor) blocking antibody (BD Pharmagen, San Diego, CA) or 200 μg rat IgG isotype control antibody (Biolegend, San Diego, CA) per mouse was i.p. injected. Injections were repeated once a week until 1 week before the mice were killed.

### 2.4 Preparation of liver macrophages for analyses of CD127 expression

Perfused mice liver tissue was homogenized, and non‐parenchymal cell pellets were obtained. Then hepatic mononuclear cells (MNCs) were isolated by using Percoll density‐gradient centrifugation. Briefly, cell pellets were resuspended in 40% Percoll (Pharmacia, Uppsala, Sweden) and layered upon 70% Percoll. The gradient was centrifuged at 2000 rpm for 20 minutes. MNCs were collected from the gradient interface, which include macrophages (F4/80^+^CD11b^+^), stellate cells (F4/80^−^CD11b^−^), lymphocytes (F4/80^−^CD11b^−^) and nature killer cell (F4/80^−^CD11b^low/hi^), but without eosinophils (F4/80^+^CD11b^+^). After F4/80 and CD11b labelling, F4/80^+^CD11b^+^ liver macrophages were purified from hepatic MNCs by flow cytometric sorting.

### 2.5 Purification of peritoneal macrophages (PMΦs) and in vitro treatment

PMΦs were widely used as substitute for liver macrophages to obtain sufficient number of pure macrophages.^18^, ^19^ Briefly, peritoneal exudate cells were obtained from normal mice by peritoneal lavage, and PMΦs were purified from peritoneal exudate cells by adherence. The purity of enriched macrophages was >99%. Purified PMΦs were treated with PBS, 10 μg/mL SEA, 10 ng/mL IL‐7 or 10 μg/mL SEA plus 10 ng/mL IL‐7 for 24 hours.

For the autophagy inhibition experiments, 2 mmol/L 3‐methyladenine (3‐MA; Sigma‐Aldrich, St. Louis, MO) was added together with the addition of PBS, SEA, IL‐7 or SEA plus IL‐7.

For AMPK activation and inhibition experiments, 10 mM metformin (Met; Sigma‐Aldrich) or 2.5 μmol/L compound C (Sigma‐Aldrich) was added 30 minutes before addition of PBS, SEA, IL‐7 or SEA plus IL‐7.

For small interfering RNA (siRNA) experiment, purified PMΦs were transfected with pooled siRNAs (Gima Biol Engineering Inc., Shanghai, China) targeting α‐subunit of AMPK or negative control siRNA using Lipofectamine 2000 (Lipo 2000; Invitrogen, Carlsbad, CA). After 48 hours, cells were further treated with PBS, SEA, IL‐7 or SEA plus IL‐7 for additional 24 hours. Pooled siRNA comprising 3 individual siRNAs.

After treatments, both suspension and adherent cells were harvested and used for flow cytometry (FCM), Western blotting or transmission electron microscopy (TEM) analysis.

### 2.6 FCM analysis

For labelling of CD127, cells were stained for 30 minutes at 4°C with anti‐CD127 antibody (eBioscience, San Diego, CA) diluted in PBS containing 1% FBS.

For the apoptosis assay, cells were resuspended in binding buffer (Lianke Biotechnology, Hangzhou, China) and incubated with Annexin V (eBioscience) and 7‐Aminoactinomycin D (7‐AAD, BD Biosciences) for 15 minutes at room temperature.

Specific labelling of intracellular LC3II was performed with a FlowCellect™ Autophagy LC3 Antibody‐based Assay Kit (Millipore Corp., Bedford, Mass) according to the instructions of the manufacturer.

All the FCM analyses were performed with the BD FACSCalibur™ flow cytometer. Results were analysed using BD CellQuest™ Pro software or FlowJo software (Treestar, Inc., San Carlos, CA). Cell sorting was performed on a BD FACSAria™ cell sorter.

### 2.7 Quantification of cell numbers

Perfused liver tissue was weighed (Mass, *M*), and hepatic MNCs were isolated as described above. The number (N) of MNCs was counted using a hemacytometer. One million MNCs were stained with anti‐F4/80 and anti‐CD11b antibodies; the percentages (*P*) of F4/80^+^CD11b^+^ macrophages were determined by FCM. The number of macrophages in per gram of liver was calculated as follows: (N×*P*)/*M*.

### 2.8 Western blotting

Whole‐cell extracts were prepared with RIPA buffer and separated by SDS‐PAGE, blotted onto nitrocellulose membranes (Whatman, Maidstone, United Kingdom) and probed with anti‐phosphorylated AMPKα (Cell Signalling Technology, Beverly, MA), anti‐AMPKα (Cell Signalling Technology) or anti‐β‐actin (Sigma‐Aldrich) antibodies, visualized using the Immobilon™ Western Chemiluminescent HRP Substrate (Millipore), detected by the Bio‐Rad Gel Doc XR System (Bio‐Rad) and quantified using the Image‐Pro Plus system (Media Cybernetics, Silver Spring, MD).

### 2.9 Reverse‐transcription PCR (RT‐PCR) and real‐time RT‐PCR

Total RNA (1 μg) extracted from tissues or cells with the RNeasy Mini Kit (Qiagen, Hilden, Germany) was reverse‐transcribed with the RevertAid™ First Strand cDNA Synthesis Kit (Fermentas, Vilnius, Lithuania).

For RT‐PCR, the resultant cDNA was amplified using Taq DNA polymerase (Promega, Madison, WI) and resolved on a 1.5% agarose gel containing ethidium bromide, detected by the Bio‐Rad Gel Doc XR System.

For real‐time RT‐PCR, SYBR Green‐based real‐time PCR was done with FastStart Universal SYBR Green Master (Rox) reagents (Roche Diagnostics, Mannheim, Germany) and an ABI PRISM 7300 Real‐Time PCR System (Applied Biosystem, Foster City, CA). Fold changes of gene expression were calculated using the 2^−ΔΔCt^ method.

### 2.10 Haematoxylin‐eosin (H&E) and Sirius Red staining

Formalin‐fixed, paraffin‐embedded liver tissues were sectioned (3 μm) and stained with H&E or Sirius Red. To perform granuloma area measurements, at least 100 granulomas with a single well‐defined egg were randomly chosen from each group at 20× objective lens, and the areas were measured using Zeiss AxioVision software (Zeiss, Oberkochen, Germany). In addition, slides stained with Sirius Red were used to measure the intensity of fibrosis. At least 10 digital images (100×) were captured from slides of each mouse. Captured images were quantified using Image‐Pro Plus system (Media Cybernetics, Silver Spring, MD). A total fibrosis density score was determined by dividing the image intensity by the image area. Intensity exclusion parameters were identical for each of the images captured.

### 2.11 TEM analysis

Cells or tissues were fixed in 2.5% glutaraldehyde in 0.1 mol/L sodium cacodylate buffer (pH 7.0) for 1 hour, post‐fixed in 1% osmium tetroxide in 0.1 mol/L cacodylate buffer for 1 hour, dehydrated with increasing concentrations of ethanol and gradually infiltrated with Araldite resin. Ultrathin sections (70‐80 nm) were stained with uranyl acetate and lead citrate and examined using a JEOL JEM 1010 transmission electron microscope (Tokyo, Japan). Autophagosomes were quantified on the basis of three independent experiments. In each experiment, 50 macrophages from each group were randomly selected for analysis.

### 2.12 Statistical analysis

Statistical comparisons between different treatments were performed by an unpaired Student's *t* test, and *P* ≤ .05 was considered statistically significant. *P* values are as indicated by asterisks: *P ≤ .05, **P ≤ .01, ***P ≤ .001.

## 3 RESULTS

### 3.1 Decreased macrophage autophagy and the increased IL‐7 expression in *S. japonicum*‐infected mice

**Figure 1:**
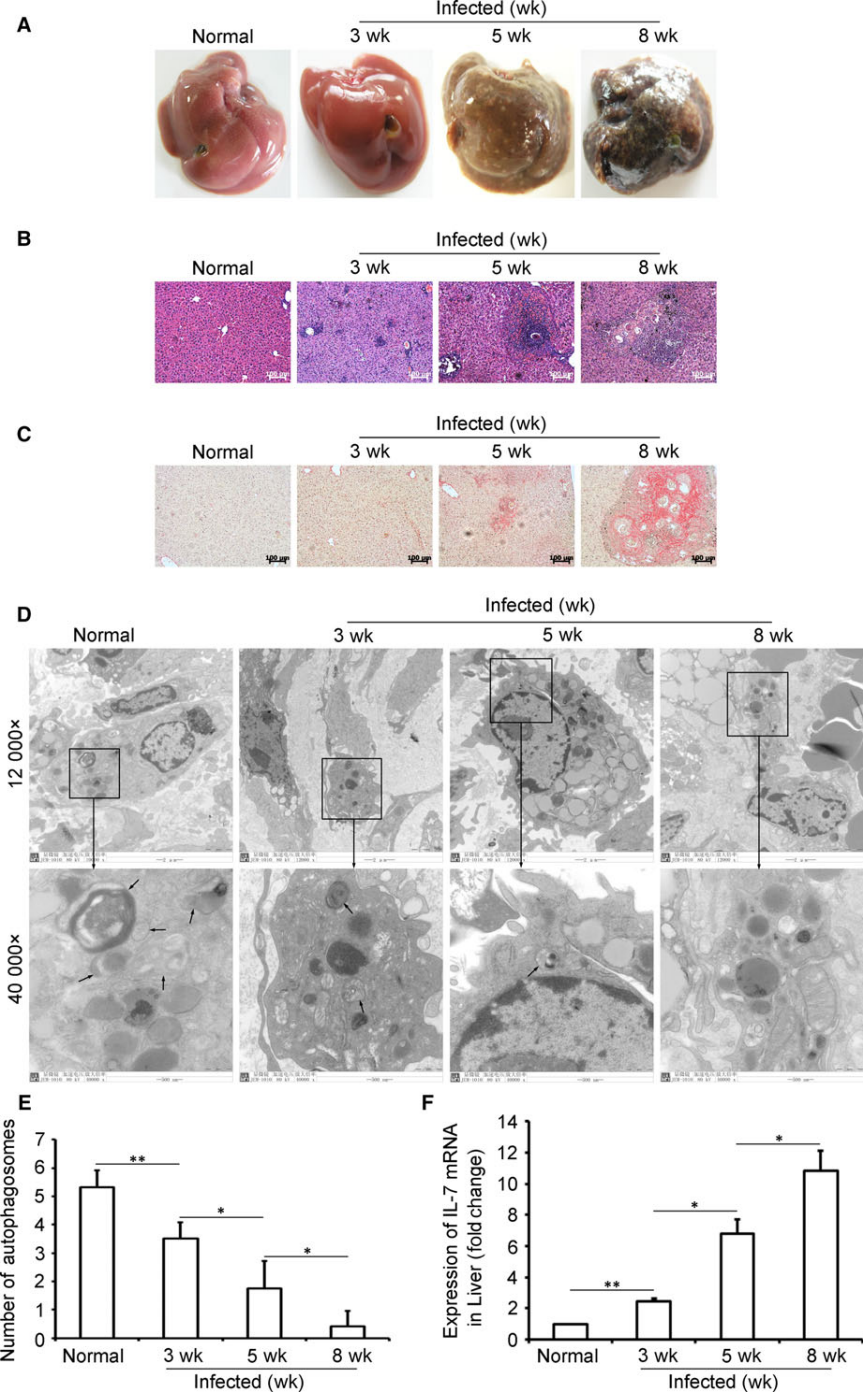
Decreased macrophage autophagy and the increased IL‐7 expression in *Schistosoma japonicum*‐infected mice. *S. japonicum*‐infected or normal control mice were killed at indicated time‐points. A, Photos of mice livers. B, H&E‐stained liver sections. The original magnification of stained liver sections was 100×. C, Sirius Red‐stained liver sections. The original magnification of stained liver sections was 100×. D and E, Autophagosomes in macrophages of liver tissue were detected by transmission electron microscope (TEM). D, Images were taken at either 12 000× or 40 000×. The 40 000× image is the enlarged image in the black frame. Black arrows in 40 000× images indicate double‐membraned autophagosomes. Images shown are representative of experiments. E, Data were means ± SD of 150 macrophages in 18 mice from three independent experiments. F, Total RNAs of livers were prepared and analysed for *Il‐7* mRNA expression by real‐time RT‐PCR. mRNA levels are relative to the normal control expression level. Data were means ± SD of 18 mice from three independent experiments. (*P ≤ .05, **P ≤ .01)

Macrophage autophagy functions to limit liver immunopathology in kinds of liver injury.^9^, ^10^, ^11^ To investigate the role of macrophage autophagy in schistosomal liver disease pathogenesis, transmission electron microscopy (TEM) analysis of autophagosomes in macrophages was performed on the sections of *S. japonicum* ‐infected mice livers. Results showed that *S. japonicum* infection induced progressive liver immunopathology (Figure 1A,B,C) parallel to the decrease in autophagosome number in liver macrophages (Figure 1D,E) and the increase in hepatic IL‐7 expression (Figure 1F). These results suggested a potential link between macrophage autophagy and IL‐7 in *S. japonicum* infection‐induced liver pathology.

### 3.2 IL‐7‐IL‐7R signalling suppresses macrophage autophagy in *S. japonicum*‐infected mice

**Figure 2:**
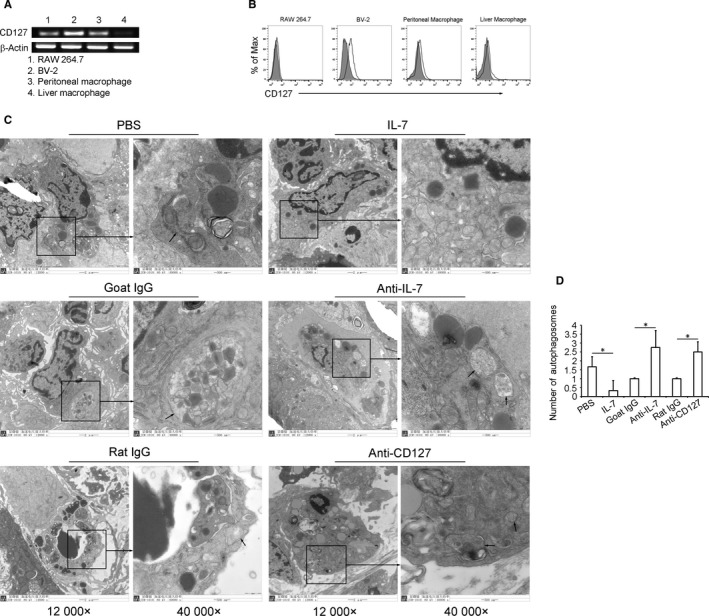
IL‐7‐IL‐7R signalling suppresses macrophage autophagy in *Schistosoma japonicum*‐infected mice. A, Total RNAs of RAW 264.7, BV‐2, purified peritoneal macrophages (PMΦs) from normal mice, and FACS sorted liver macrophages from normal mice were analysed for *Cd127* mRNA expression by RT‐PCR followed by agarose gel electrophoresis. B, Expression of CD127 was measured by FCM. Grey‐filled lines in FCM plots indicate the isotype control, and unfilled lines indicate the CD127‐stained cells. Agarose gel and FCM plots are representative of three independent experiments. *S. japonicum*‐infected mice were injected with PBS, IL‐7, goat IgG isotype antibody, anti‐IL‐7 neutralizing antibody, rat IgG isotype antibody or anti‐CD127 blocking antibody as described in Methods. Autophagosomes in macrophages of liver tissue were detected by TEM. C, Images were taken at either 12 000× or 40 000×. The 40 000× image is the enlarged image in the black frame. Black arrows in 40 000× images indicate double‐membraned autophagosomes. Images shown are representative of experiments. D, Data were means ± SD of 150 macrophages in 18 mice from three independent experiments. (*P ≤ .05)

For the first time, our results showed that the specific chain of IL‐7R (CD127) was expressed in macrophages (Figure 2A,B). To determine the role of IL‐7‐IL‐7R signalling in macrophage autophagy in *S. japonicum*‐infected mice liver, IL‐7, anti–IL‐7 neutralizing antibody or anti‐CD127 blocking antibody were injected into mice after 3 weeks of schistosome infection. Results showed that compared to PBS control group, injection of IL‐7 significantly decreased the number of autophagosomes in macrophage (Figure 2C,D). Conversely, anti‐IL‐7 neutralizing or anti‐CD127 blocking antibody treatment increased the number of autophagosomes in macrophage (Figure 2C,D). These results suggested an anti‐autophagic role of IL‐7 in macrophage.

### 3.3 IL‐7 directly suppresses macrophage autophagy in vitro

**Figure 3:**
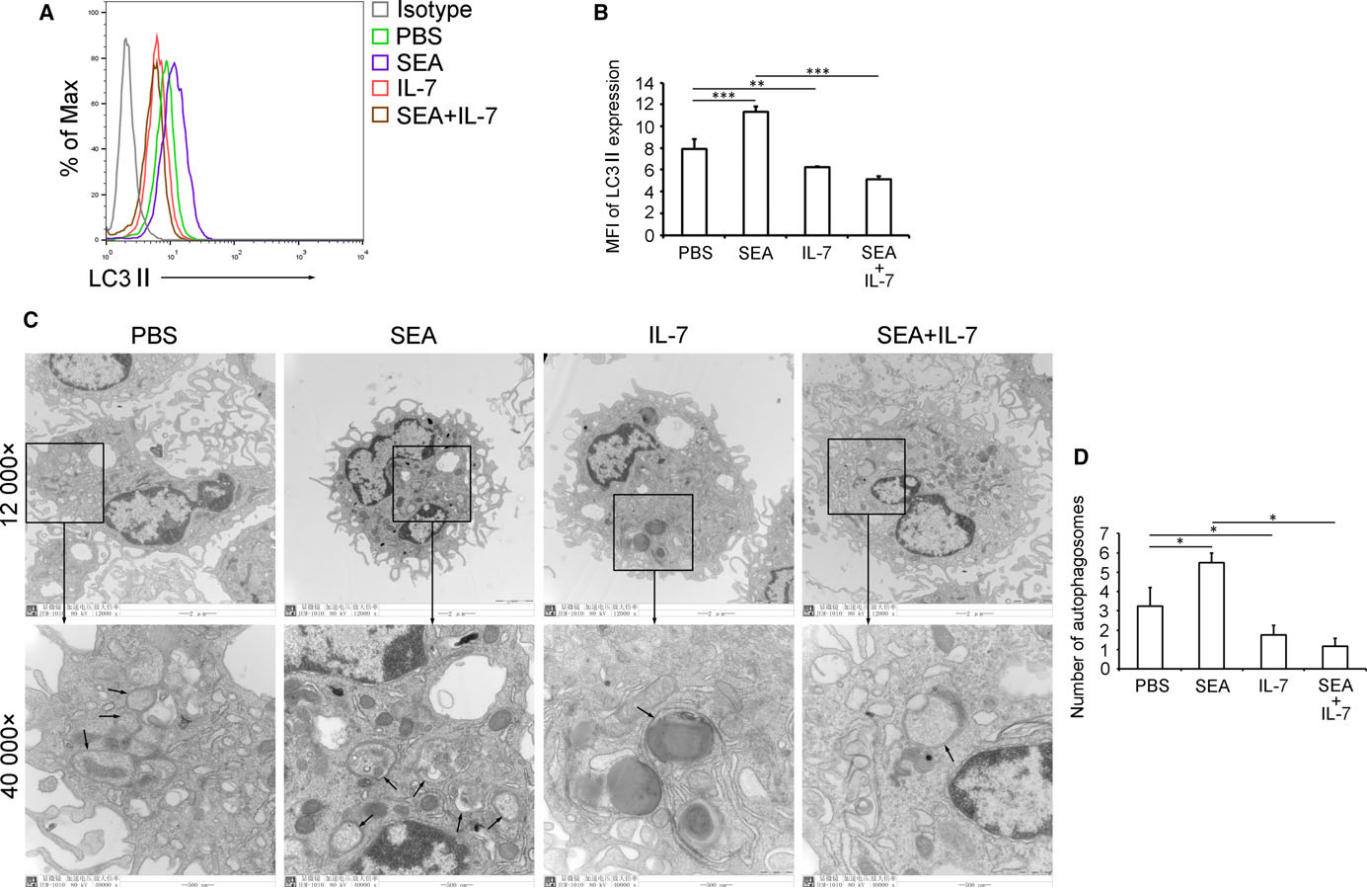
IL‐7 directly suppresses macrophage autophagy in vitro. Purified PMΦs from normal mice were treated with PBS, SEA, IL‐7 or SEA plus IL‐7 for 24 h. A and B, LC3II expression was detected by FCM. A, FCM plot is representative of experiments. B, Data were mean fluorescence intensity (MFI) ± SD of three independent experiments. C and D, Autophagosomes were detected by TEM. C, Images were taken at either 12 000× or 40 000×. The 40 000× image is the enlarged image in the black frame. Black arrows in 40 000× images indicate double‐membraned autophagosomes. Images shown are representative of experiments. D, Data were means ± SD of 150 macrophages from three independent experiments. (*P ≤ .05, **P ≤ .01, ***P ≤ .001). SEA, soluble egg antigen; PMΦ, peritoneal macrophages; TEM, transmission electron microscope.

To confirm the role of IL‐7 in macrophage autophagy suppression, purified PMΦs from normal mice (Figure S1) were treated with IL‐7 and/or SEA, a mixture of the main schistosome antigens that induce pathologic liver granulomatous responses. Results showed SEA significantly induced but IL‐7 dramatically suppressed the level of autophagy marker LC3II in macrophages (Figure 3A,B). These results were further confirmed by the TEM analysis of autophagosomes in macrophages (Figure 3C,D).

Taken together with the in vivo observation that anti‐CD127 blocking antibody efficiently inhibited the anti‐autophagic function of IL‐7 on macrophage autophagy, these findings implied that in schistosome‐infected mice, regulation of the macrophage autophagy by IL‐7‐IL‐7R axis may be one of the mechanisms underlying the control of liver pathology.

### 3.4 Anti‐autophagic IL‐7 enhances pathology in *S. japonicum*‐infected murine livers

**Figure 4:**
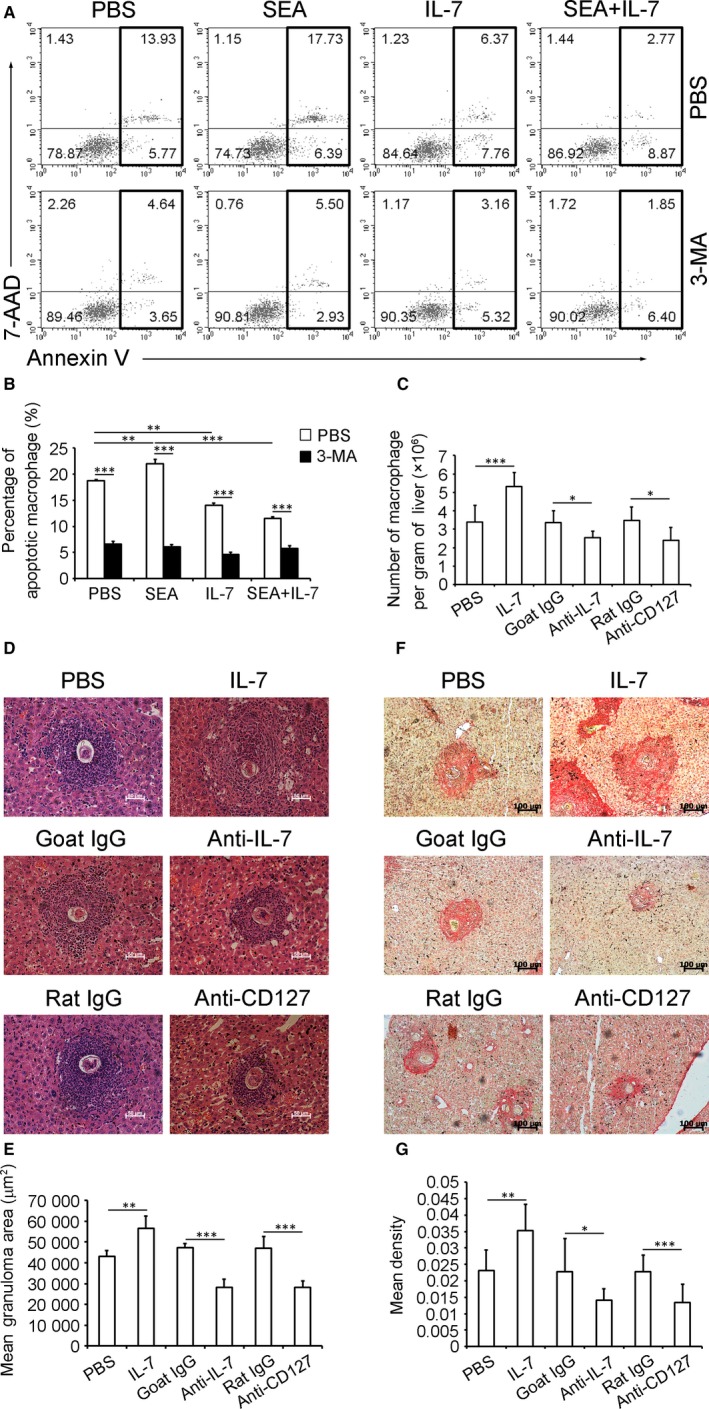
Anti‐autophagic IL‐7 enhances pathology in *S. japonicum*‐infected murine livers. Purified PMΦs from normal mice were treated with 3‐MA or an equal volume of PBS together with PBS, SEA, IL‐7 or SEA plus IL‐7 for 24 h. The apoptotic macrophages were determined by FCM. A, FCM plots are representative of experiments. B, Data were means ± SD of three independent experiments. Mice were infected and treated as described in Figure 2C legend. C, Liver MNCs were isolated and stained for macrophages analysis by FCM and numbers of macrophages in per gram of liver were calculated as described in Methods. Data were means ± SD of 18 mice from three independent experiments. D and E, H&E staining was performed in liver sections, and areas of single egg granulomas were measured. D, The original magnification of stained liver sections was 200×. Photos are representative of experiments. E, Data were means ± SD of 18 mice from three independent experiments. F and G, Sirius Red staining was performed in liver sections, and quantification of fibrosis was performed. F, The original magnification of stained liver sections was 100×. Photos are representative of experiments. G, Data were means ± SD of 18 mice from three independent experiments. (*P ≤ .05, **P ≤ .01, ***P ≤ .001)

Studies suggest that autophagy may contribute to apoptotic program.^20^, ^21^ We used 3‐methyladenine (3‐MA), an inhibitor of autophagy, to determine whether inhibition of autophagy alters macrophage apoptosis. Results showed that SEA treatment significantly increased but IL‐7 significantly reduced the percentage of apoptotic (Annexin V^+^) PMΦs (Figure 4A,B). However, 3‐MA significantly decreased macrophage apoptosis in all groups, including the significant decrease in the pro‐apoptotic effect of SEA and the enhancement of the anti‐apoptotic effect of IL‐7 (Figure 4A,B).

Macrophage apoptosis contributes to cell loss and thus is critical for the regulation of efficient resolution of inflammation.^22^, ^23^ We further determined the role of IL‐7‐IL‐7R axis in the regulation of macrophage number and liver pathology (granulomatous inflammation and subsequent fibrosis) in *S. japonicum*‐infected mice. Results showed that IL‐7 treatment significantly increased the number of liver macrophages (Figure 4C) and enhanced the granuloma development (Figure 4D,E) and collagen deposits (Figure 4F,G), but anti‐IL‐7 or anti‐CD127 antibody treatment significantly decreased the number of liver macrophages (Figure 4C) and attenuated the granuloma development (Figure 4D,E) and collagen deposits (Figure 4F,G). Of note, neither the morphology of liver macrophages (Figure S2) nor the development and fecundity of parasites (Figure S3) was significantly affected when mice were injected with IL‐7, anti‐IL‐7 neutralizing antibody or anti‐CD127 blocking antibody 3 weeks after *S. japonicum* infection, indicating that IL‐7 signalling contributes to the liver pathology in schistosomiasis via regulation of host immune responses instead of affecting the development and fecundity of parasites per se.

### 3.5 IL‐7 suppresses macrophage autophagy via AMPK

**Figure 5:**
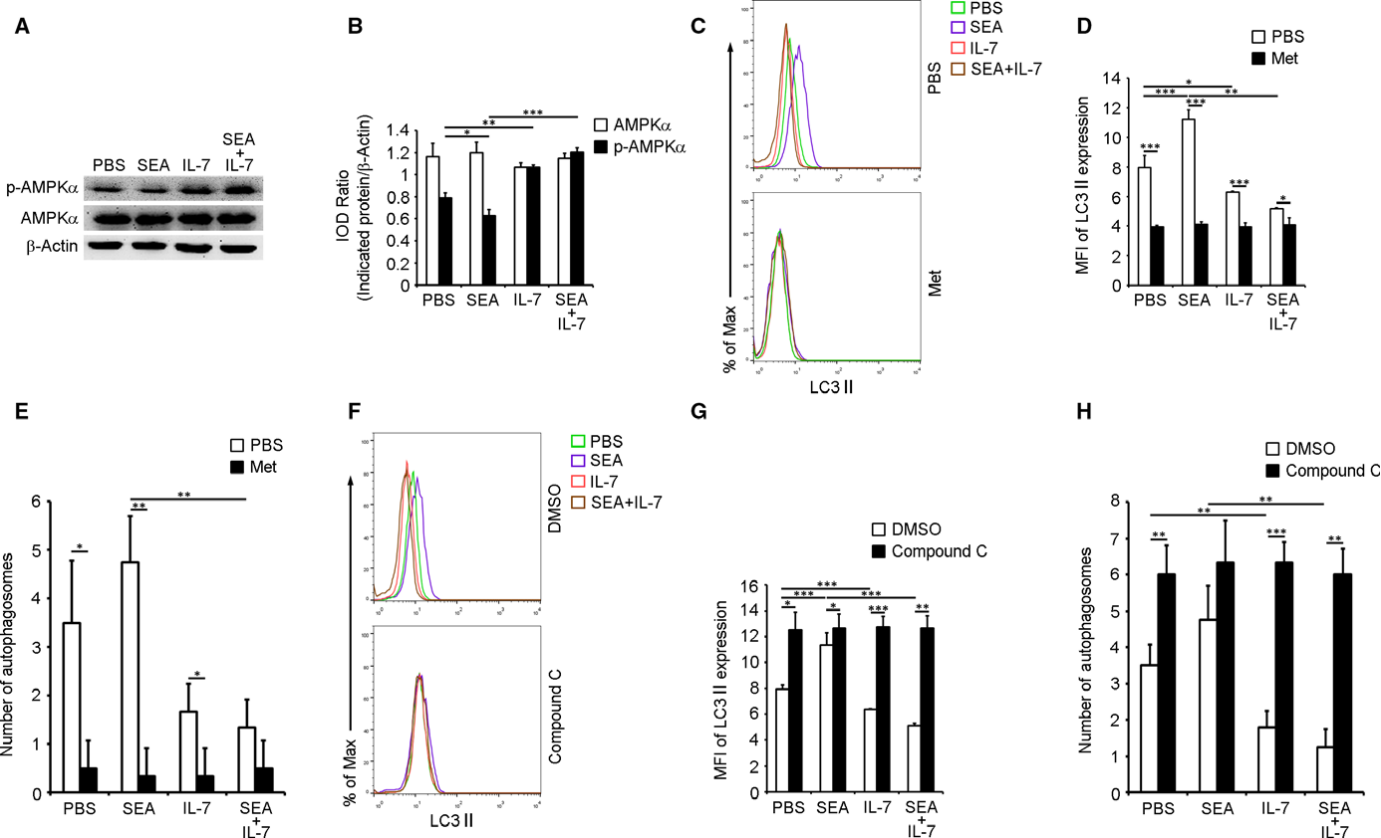
IL‐7 suppresses macrophage autophagy via AMP‐activated protein kinase (AMPK). Purified PMΦs from normal mice were treated as described in Figure 3A legend. A and B, Phosphorylated AMPKα and total AMPKα were detected by Western blotting. Scanning densitometric analysis was performed, and the integrated optical density (IOD) of each band was normalized to corresponding β‐actin. A, Blots shown are representative of experiments. B, Data were means ± SD of three independent experiments. C‐H, Purified PMΦs from normal mice were pre‐treated with Met or compound C or an equal volume of solvent (PBS or DMSO) for 30 min; then cells were treated with PBS, SEA, IL‐7 or SEA plus IL‐7 for another 24 h. C, D, F and G, LC3II expression was detected by FCM. C and F, FCM plots are representative of experiments. D and G, Data were MFI ± SD of three independent experiments. E and H, Autophagosomes were detected by TEM. Data were means ± SD of 150 macrophages from three independent experiments. (*P ≤ 0.05, **P ≤ 0.01, ***P ≤ 0.001)

Besides the role of AMPK as a master regulator of cellular metabolism, emerging evidence implicates its role in the prevention of autophagy.^24^, ^25^ Our results in Figure 5A,B showed that SEA significantly suppressed, while IL‐7 significantly induced the activation of AMPK in purified PMΦs. Metformin (Met) and compound C were widely used as pharmacological activator or inhibitor of AMPK, respectively.^26^, ^27^ Results showed that Met significantly decreased levels of LC3II expression (Figure 5C,D) and autophagosomes (Figures 5E and S4) in all groups, including the significant decrease in the pro‐autophagic effect of SEA and the enhancement of the anti‐autophagic effect of IL‐7. However, compound C significantly increased levels of LC3II expression (Figure 5F,G) and autophagosomes (Figures 5H and S5) in all groups. These results suggested that SEA and IL‐7 might promote or prevent macrophage autophagy by inhibiting and stimulating AMPK activation, respectively.

**Figure 6:**
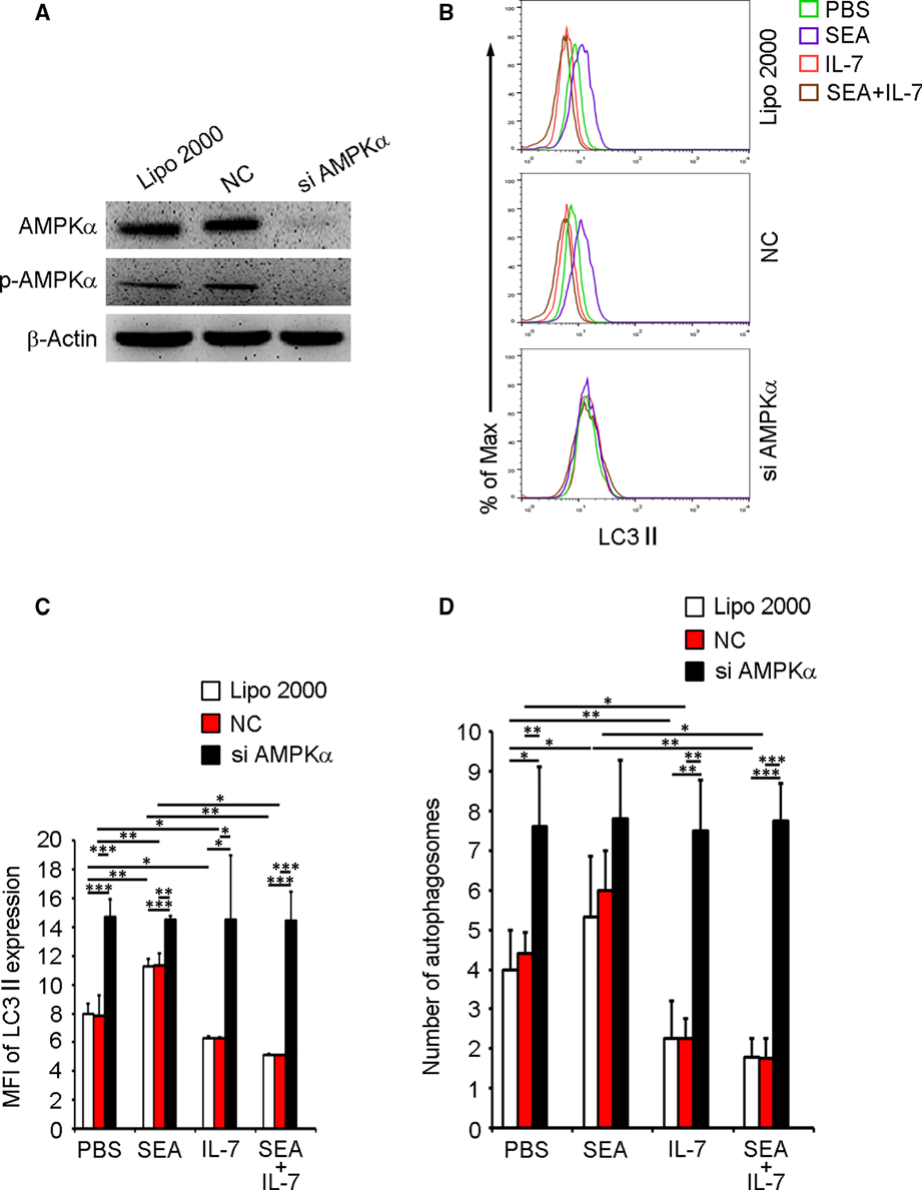
Knock‐down of AMPKα induces macrophage autophagy. Purified PMΦs from normal mice were transfected with pooled siRNAs targeting α‐subunit of AMPK or negative control (NC) siRNA using Lipofectamine 2000 (Lipo 2000) as described in Methods. 48 h later, (A) total AMPKα and phosphorylated AMPKα were detected by Western blotting. Blots shown are representative of three independent experiments. B‐D, cells were treated with PBS, SEA, IL‐7 or SEA plus IL‐7 for another 24 h. B and C, LC3II expression was detected by FCM. B, FCM plots are representative of experiments. C, Data were MFI ± SD of three independent experiments. D, Autophagosomes were detected by TEM. Data were means ± SD of 150 macrophages from three independent experiments. (*P ≤ .05, **P ≤ .01, ***P ≤ .001)

To further confirm that AMPK is required for the anti‐autophagic effects of IL‐7, expression of AMPK was down‐modulated using α‐subunit‐targeted siRNA. Immunoblot analysis revealed that siRNA targeting α‐subunit dramatically reduced the total and phosphorylated AMPKα levels (Figure 6A). Meanwhile, siRNA treatment also significantly increased LC3II expression (Figure 6B,C) and autophagosomes (Figures 6D and S6) in all groups. Taken together, these results indicate that IL‐7 protects macrophages from autophagy via AMPK.

## 4 DISCUSSION

In hosts infected with *S. japonicum* or *mansoni*, parasite eggs are trapped in hosts’ liver and induce severe liver granulomatous responses, which subsequently lead to liver fibrosis, circulatory impairment or even death.^2^, ^3^ Macrophage is one of the critical participants in development of the liver immunopathology initiated by parasite eggs in host liver.^2^, ^8^ In this study, we showed for the first time that IL‐7 induced in *S. japonicum* infection suppressed AMPK‐dependent macrophage autophagy and enhanced liver immunopathology.

Consistent with previous study,^28^ here we showed that the expression of IL‐7 significantly increased after transcutaneous infection of mice with schistosome cercariae, correlating with the enhancement of the immunopathology in the liver. Considering we found that IL‐7R was expressed on macrophages and demonstrated decreased macrophage autophagy paralleled with increased IL‐7 expression in *S. japonicum*‐infected mice, it is reasonable for us to postulate that IL‐7 could play a role to suppress macrophage autophagy, resulting in exacerbation of liver pathology. Based on our results, we for the first time provided in vivo and in vitro evidences to demonstrate that IL‐7 protects macrophages from autophagy by binding to IL‐7R. Considering that macrophages not only consist up to 30% of the granuloma cells but also act as critical antigen‐presenting cells in granulomatous inflammation, our results may suggest a novel role for IL‐7 in macrophage autophagy and granulomatous inflammation regulation and indicate that increase in macrophage autophagy by the decrease in IL‐7 could be one of the important interventions to control the liver immunopathology after schistosome infection.

Our data showed that macrophage apoptosis was inhibited by the autophagy inhibitor 3‐MA. This result is in line with previous studies. For example, in hepatoma cells, inhibition of autophagy via BafA 1 or Atg5 siRNA decreases ASPP2 (apoptosis‐stimulating protein of p53‐2)‐induced apoptosis.^20^ In primary cortical neurons, inhibition of autophagy by 3‐MA, or lentivirally delivered shRNAs against Atg5 and Atg7, strongly reduced the staurosporine‐induced apoptosis.^21^ As macrophage is a major component of granuloma cells, macrophage autophagy might functions to limit liver pathology by its pro‐apoptotic activity. And, we have demonstrated that IL‐7 suppresses macrophage autophagy and exacerbated liver pathology simultaneously during *S. japonicum* infection, and we speculate that IL‐7 might enhance liver immunopathology through a mechanism involving inhibition of the autophagic apoptosis in macrophage. Meanwhile, we also understand that except for by suppressing autophagy of macrophages in liver, IL‐7 may also contribute to the regulation of the liver immunopathology by its anti‐apoptotic effect on T cells.

To date, mechanisms by which IL‐7 regulates autophagy remain poorly investigated. In this study, we identified AMPK as a novel target downstream of IL‐7 signalling to suppress macrophage autophagy. We postulate that AMPK inhibits autophagy via inactivation of the mTOR pathway through TSC1/2‐complex activation, based on the finding that mTOR promotes autophagy by inhibition of Akt, a well‐known inhibitor of autophagy, in human cancer cells.^29^ However, more studies need to be done in the future to further elucidate details of the mechanisms involved in the regulation of AMPK activation by IL‐7.

To sum up, our study reports a novel role of IL‐7 in regulation of macrophage autopahgy and identifies AMPK as a downstream target of IL‐7‐IL‐7R signalling, and suggests manipulation of macrophage autophagy through IL‐7‐IL‐7R pathway as a possible therapeutic option for schistosomiasis.

## CONFLICT OF INTEREST

The authors confirm that there are no conflicts of interest.
