# Supplementary figures and images for: IL‐7 suppresses macrophage autophagy and promotes liver pathology in Schistosoma japonicum‐infected mice
Source: J Cell Mol Med. 2018 Mar 22;22(7):3353–63. doi: 10.1111/jcmm.13610 (PMC6010884; doi:10.1111/jcmm.13610)

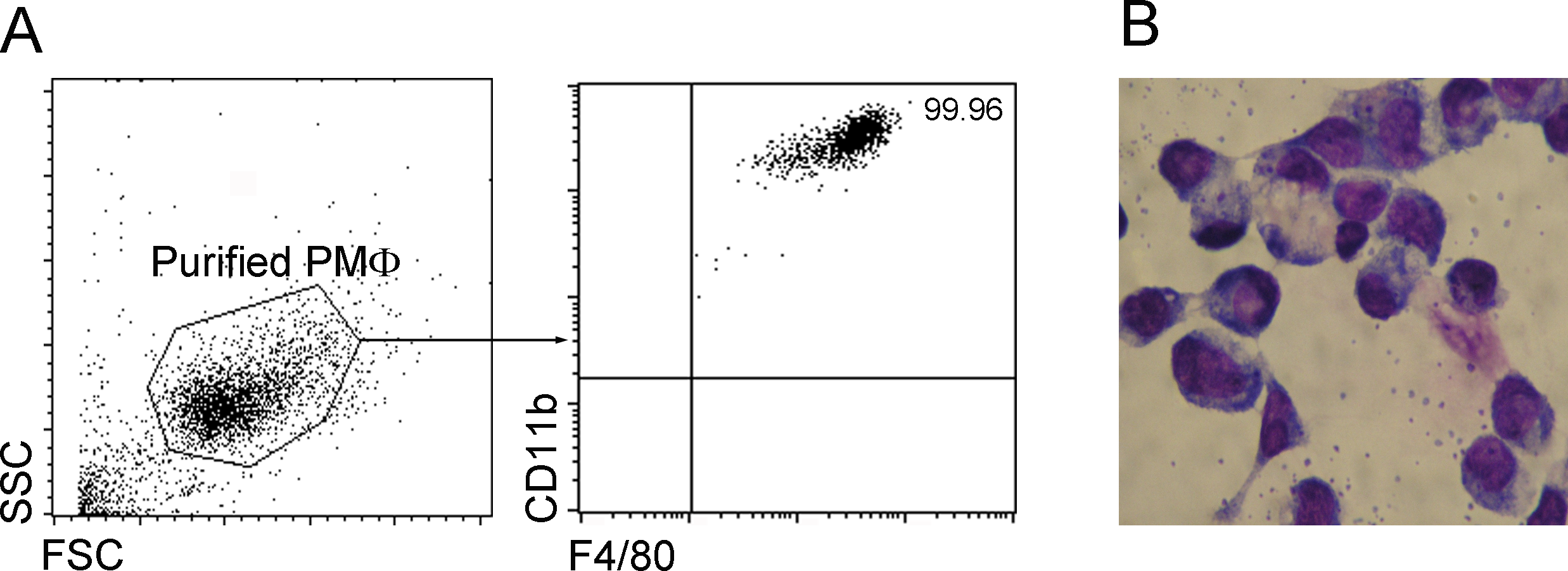

Supplement: Supplementary file 1 [file JCMM-22-3353-s001.tiff]

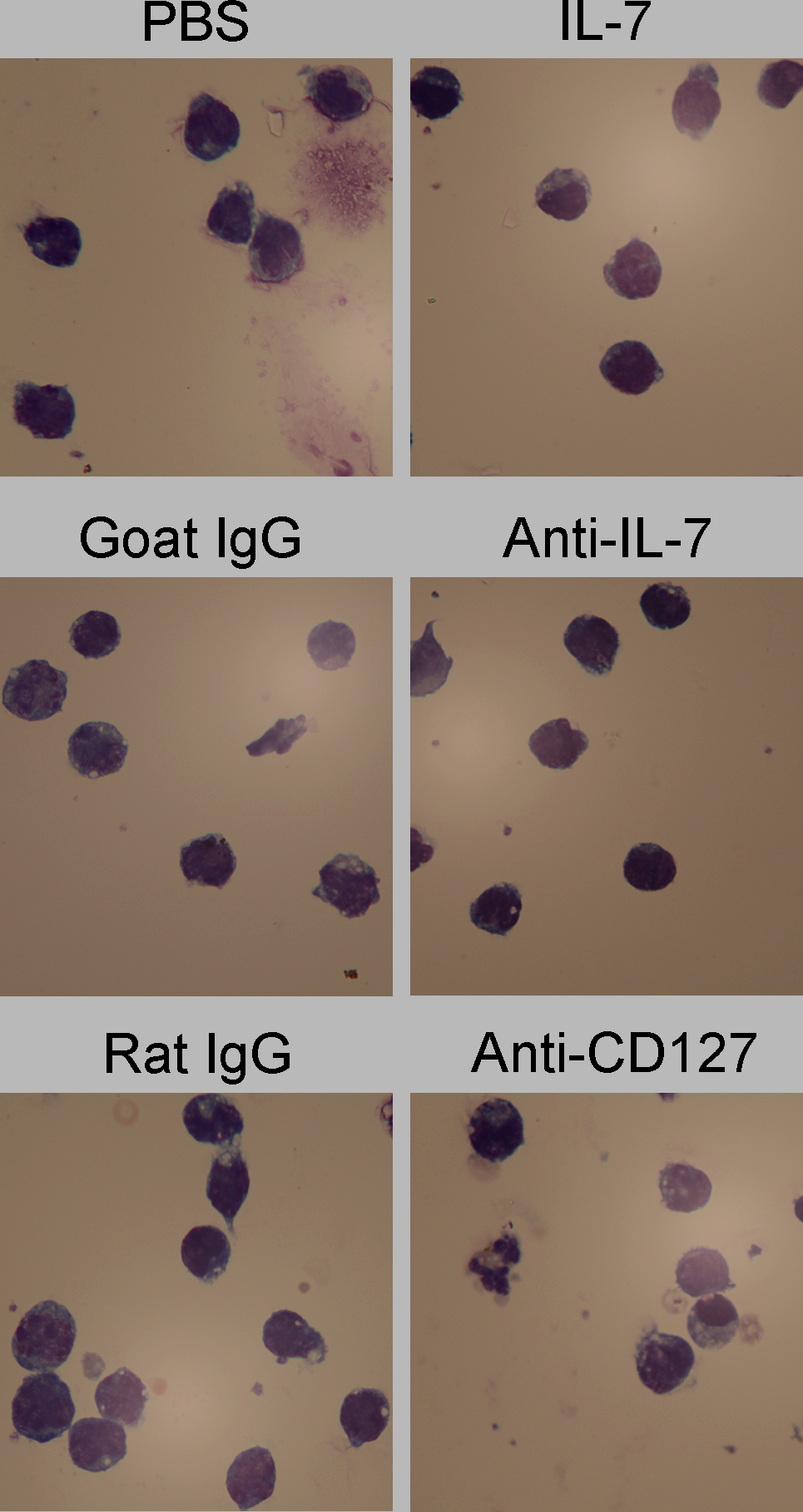

Supplement: Supplementary file 2 [file JCMM-22-3353-s002.tiff]

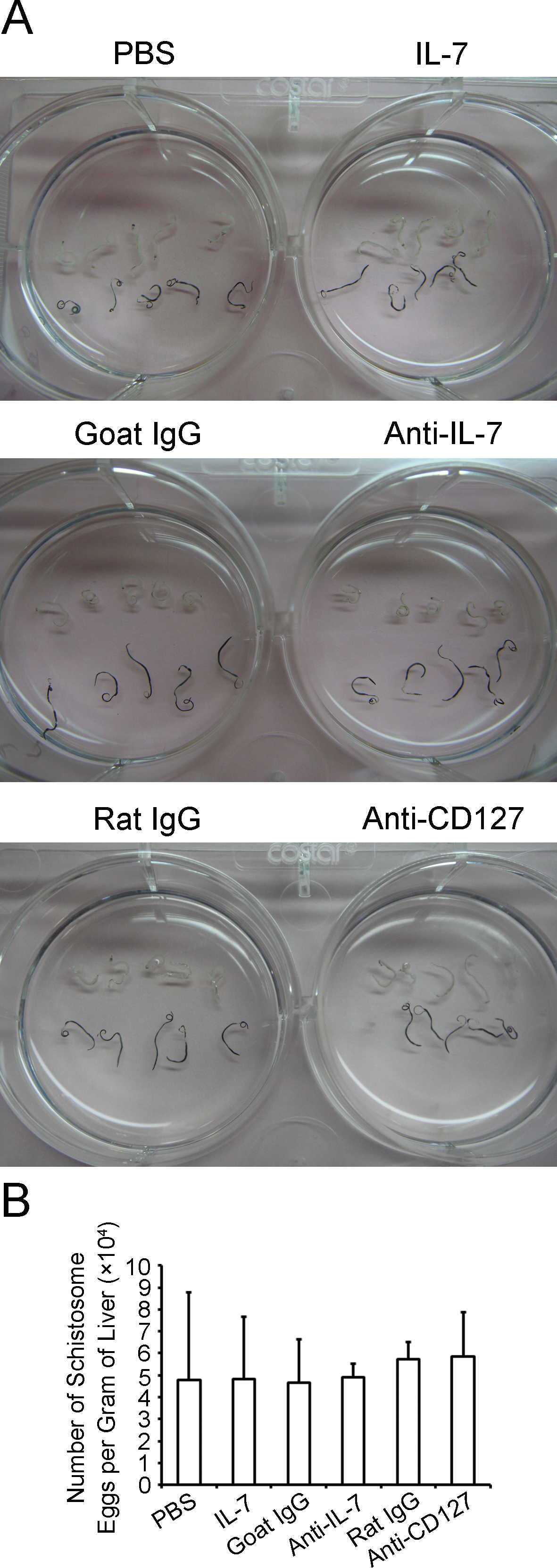

Supplement: Supplementary file 3 [file JCMM-22-3353-s003.tiff]

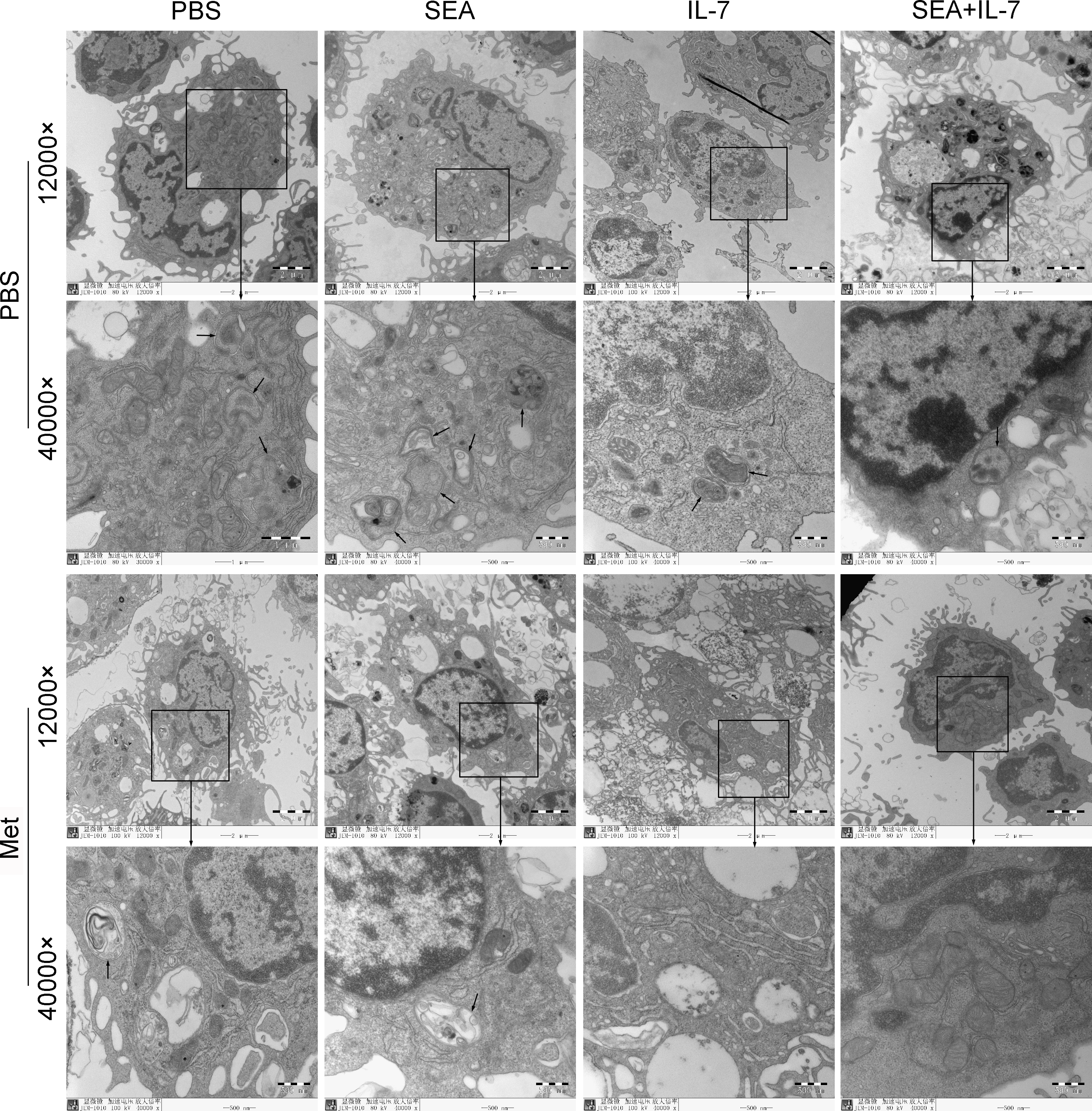

Supplement: Supplementary file 4 [file JCMM-22-3353-s004.tiff]

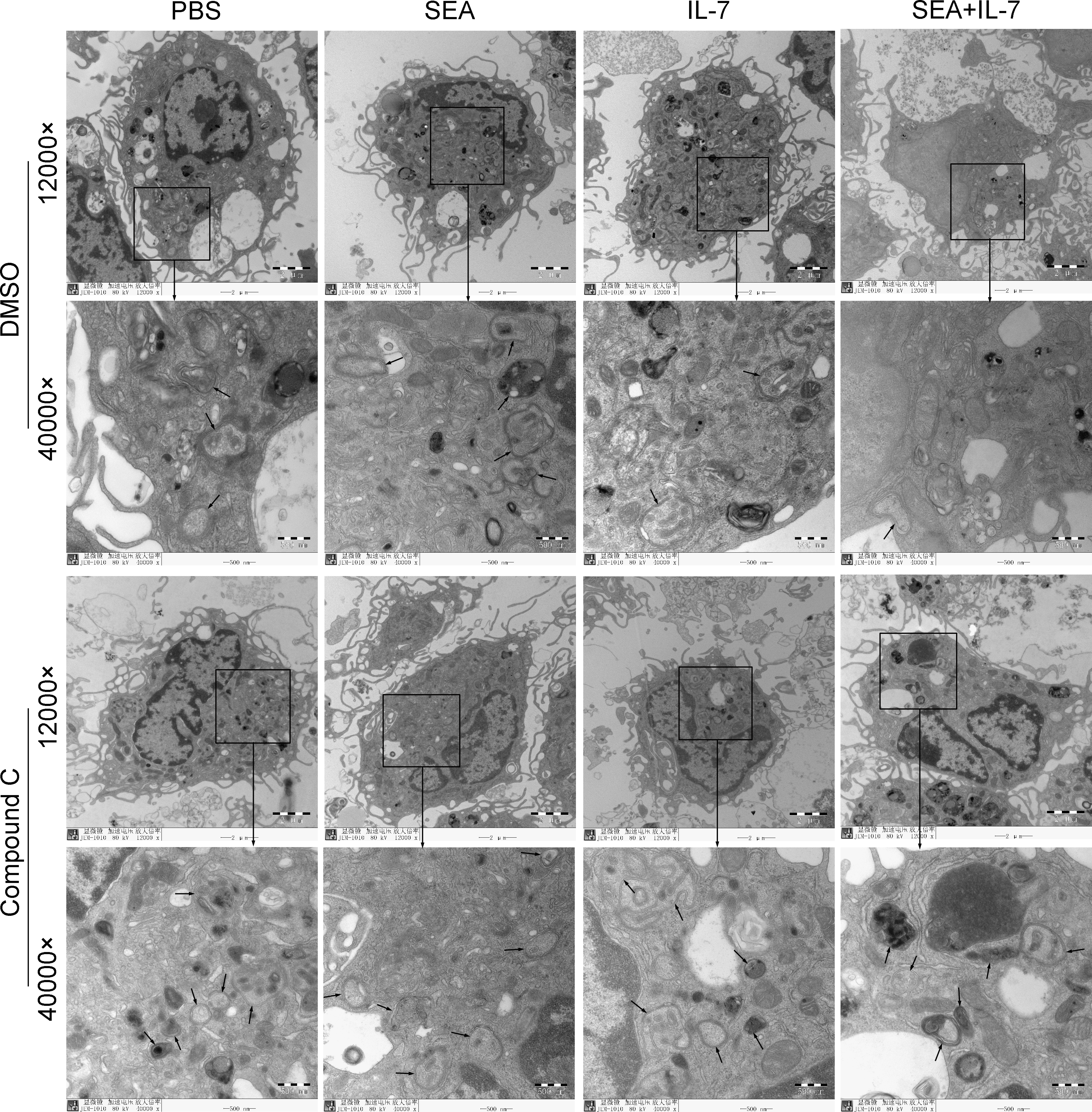

Supplement: Supplementary file 5 [file JCMM-22-3353-s005.tiff]

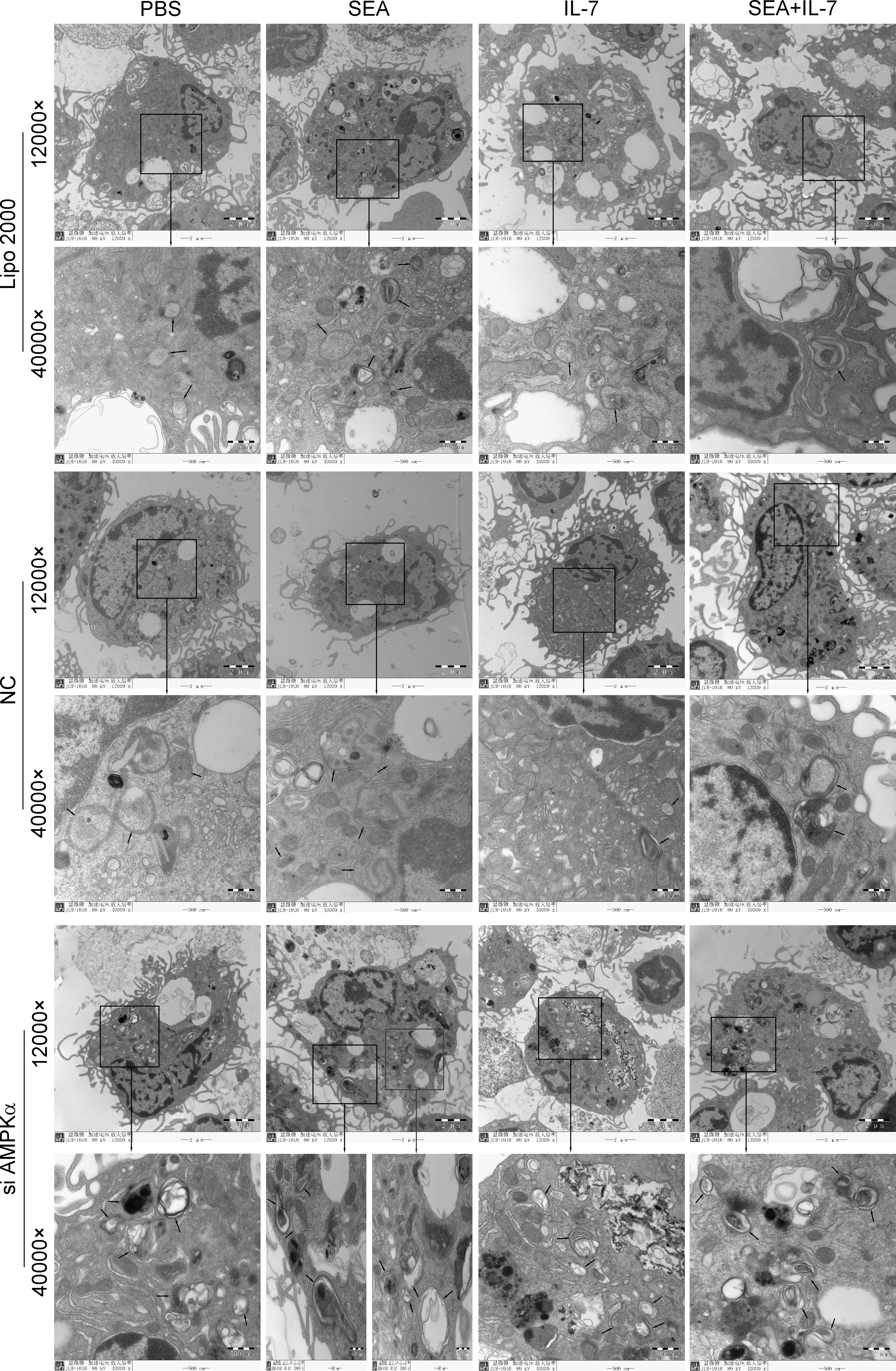

Supplement: Supplementary file 6 [file JCMM-22-3353-s006.tiff]
