# Supplementary material for: IL‐7 suppresses macrophage autophagy and promotes liver pathology in Schistosoma japonicum‐infected mice
Source: J Cell Mol Med. 2018 Mar 22;22(7):3353–63. doi: 10.1111/jcmm.13610 (PMC6010884; doi:10.1111/jcmm.13610)
Supplement: Supplementary file 7 [file JCMM-22-3353-s007.docx]

**Supporting Information**

**IL-7 Suppresses Macrophage Autophagy and Promotes Liver Pathology in *Schistosoma japonicum*-Infected Mice**

Jifeng Zhu, Weiwei Zhang, Lina Zhang, Lei Xu, Xiaojun Chen, Sha Zhou, Zhipeng Xu, Ming Xiao, Hui Bai, Feng Liu, Chuan Su*

**Materials and Methods**

**Morphology staining determination**

Morphological analysis was performed using the Wright-Giemsa stain (Sigma-Aldrich, St. Louis, MO). The slides were observed and photographed under a light microscope at 100× objective lens.

**Flow cytometry (FCM) analysis**

For labeling of surface molecules including F4/80, CD11b, cells were stained for 30 min at 4^°^C with antibodies diluted in PBS containing 1% FBS.

Fluorescence labeled anti-F4/80, anti-CD11b antibodies were from eBioscience (San Diego, CA). All the FCM analyses were performed with the BD  FACSCalibur™ flow cytometer. Results were analyzed using BD CellQuest^TM^ Pro software.

**Quantification of Schistosome eggs**

Liver egg counts were performed on livers digested in 5% KOH (potassium hydroxide) solution according the method of Cheever [38].

**PCR primers used in this study**

Reverse-transcription PCR (RT-PCR) primers

| Primer name | Primer sequence (5′-3′) | |
| --- | --- | --- |
| *Cd127* | forward | GCCTGTCACATCATCTGAGTGCC |
|  | reverse | CAGGAGGCATCCAGGAACTTCTG |
| *β-actin* | forward | TTGGGTATGGAATCCTGTG |
|  | reverse | CATCGTACTCCTGCTTGC |

Real-time RT-PCR primers

| Primer name | Primer sequence (5′-3′) | |
| --- | --- | --- |
| *Il-7* | forward | GGAATTCCTCCACTGATC |
|  | reverse | TGTCTTTAATGTGGCACTCAGATGAT |
| *Gapdh* | forward | GGTGAAGGTC GGTGTGAACG |
|  | reverse | ACCATGTAGTTGAGGTCAATGAAGG |

**SiRNA sequences used in this study**

| SiRNA | Sequence (5′-3′) | |
| --- | --- | --- |
| *Ampkα* siRNA-1 | forward | GUGGAUAGUAGGACUUACUTT |
|  | reverse | AGUAAGUCCUACUAUCCACTT |
| *Ampkα* siRNA-2 | forward | GGACCCAUCUUAUAGUUCATT |
| *Ampkα* siRNA-3  Negative control | reverse  forward  reverse  forward  reverse | GGACCCAUCUUAUAGUUCATT  GUGGUCCACAGAGAUUUGATT  UCAAAUCUCUGUGGACCACTT  UUCUCCGAACGUGUCACGUTT  ACGUGACACGUUCGGAGAATT |

**Figure legends**

**Fig. S1**

Quality controls for macrophage. PMΦs were purified from peritoneal exudate cells by adherence as described in the Materials and Methods. (**A**) The purity of enriched PMΦ was examined by F4/80 and CD11b staining using FCM. (**B**) Morphological analysis was performed on adherent PMΦ using the Wright-Giemsa stain. The original magnification of stained slides was 1000×. Images shown are representative of three independent experiments.

**Fig. S2**

Alteration of IL-7 levels 3 weeks after schistosome infection did not affect liver macrophage morphology. *S. japonicum*-infected mice were injected with PBS, IL-7, goat IgG isotype control antibody, anti-IL-7 neutralizing antibody, rat IgG isotype control antibody, or anti-CD127 blocking antibody as described in Materials and Methods. Liver MNCs were prepared and F4/80^+^CD11b^+^MNCs were FACS-sorted, then morphological analysis was performed on F4/80^+^CD11b^+^MNCs by cytospin preparation using the Wright-Giemsa stain. The original magnification of stained slides was 1000×. Images shown are representative of three independent experiments.

**Fig. S3**

Alteration of IL-7 levels 3 weeks after schistosome infection did not affect worm development or oviposition in infected mice. Mice were infected and treated as described in Fig. S2 legend. (**A**) Male (n = 5) and female (n = 5) worms from each treatment mice were randomly picked and put into a 6-well plate. Photos are representative of experiments. (**B**) The number of eggs in per gram of liver was calculated as described in the Materials and Methods. Data were means ± SD of 18 mice from three independent experiments.

**Fig. S4**

Metformin (Met) decreased macrophage autophagy. Purified PMΦs from normal mice were pretreated with Met for 30min, an equal volume of PBS served as control, then cells were treated with PBS, SEA, IL-7, or SEA plus IL-7 for another 24 h. Autophagosomes were detected by TEM. Images were taken at either 12000× or 40000×. The 40000× image is the enlarged image in the black frame. Black arrows in 40000× images indicate double-membraned autophagosomes. Images shown are representative of three independent experiments.

**Fig. S5**

Compound C induced macrophage autophagy. Purified PMΦs from normal mice were pretreated with compound C for 30min, an equal volume of DMSO served as control, then cells were treated with PBS, SEA, IL-7, or SEA plus IL-7 for another 24 h. Autophagosomes were detected by TEM. Images were taken at either 12000× or 40000×. The 40000× image is the enlarged image in the black frame. Black arrows in 40000× images indicate double-membraned autophagosomes. Images shown are representative of three independent experiments.

**Fig. S6**

SiRNA-mediated suppression of AMPK expression increased macrophage autophagy. Purified PMΦs from normal mice were transfected with pooled siRNAs targeting α subunit of AMPK or negative control (NC) siRNA using Lipofectamine 2000 (Lipo 2000). After 48h, cells were treated with PBS, SEA, IL-7, or SEA plus IL-7 for another 24 h. Autophagosomes were detected by TEM. Images were taken at either 12000× or 40000×. The 40000× image is the enlarged image in the black/grey frame. Black arrows in 40000× images indicate double-membraned autophagosomes. Images shown are representative of three independent experiments.
